# Role of Pharmacogenetics in the Treatment of Acute Myeloid Leukemia: Systematic Review and Future Perspectives

**DOI:** 10.3390/pharmaceutics14030559

**Published:** 2022-03-03

**Authors:** Álvaro Pinto-Merino, Jorge Labrador, Pablo Zubiaur, Raquel Alcaraz, María José Herrero, Pau Montesinos, Francisco Abad-Santos, Miriam Saiz-Rodríguez

**Affiliations:** 1 Department of Health Sciences, University of Burgos, 09001 Burgos, Spain; apinto@ucm.es; 2 Research Unit, Fundación Burgos por la Investigación de la Salud (FBIS), Hospital Universitario de Burgos, 09006 Burgos, Spain; jlabradorg@saludcastillayleon.es (J.L.); ralcaraz@hubu.es (R.A.); 3 Haematology Department, Hospital Universitario de Burgos, 09006 Burgos, Spain; 4 Facultad de Ciencias de la Salud, Universidad Isabel I, 09003 Burgos, Spain; 5 Centro de Investigación Biomédica en Red de Enfermedades Hepáticas y Digestivas (CIBERehd), 28029 Madrid, Spain; pablo.zubiaur@salud.madrid.org (P.Z.); francisco.abad@salud.madrid.org (F.A.-S.); 6 Clinical Pharmacology Department, Hospital Universitario de La Princesa, Instituto de Investigación Sanitaria La Princesa (IP), Universidad Autónoma de Madrid (UAM), 28006 Madrid, Spain; 7 Pharmacogenetics Department, Hospital Universitari i Politècnic La Fe, 46026 Valencia, Spain; maria.jose.herrero@uv.es; 8 Haematology Department, Hospital Universitari i Politècnic La Fe, 46026 Valencia, Spain; montesinos_pau@gva.es

**Keywords:** acute myeloid leukemia, pharmacogenetics, cytarabine, anthracyclines, FLT3 inhibitors, IDH inhibitors, CD33 inhibitors, hypomethylating agents, BCL2 inhibitors

## Abstract

Acute myeloid leukemia (AML) is a heterogeneous disease characterized by remarkable toxicity and great variability in response to treatment. Plenteous pharmacogenetic studies have already been published for classical therapies, such as cytarabine or anthracyclines, but such studies remain scarce for newer drugs. There is evidence of the relevance of polymorphisms in response to treatment, although most studies have limitations in terms of cohort size or standardization of results. The different responses associated with genetic variability include both increased drug efficacy and toxicity and decreased response or resistance to treatment. A broad pharmacogenetic understanding may be useful in the design of dosing strategies and treatment guidelines. The aim of this study is to perform a review of the available publications and evidence related to the pharmacogenetics of AML, compiling those studies that may be useful in optimizing drug administration.

## 1. Acute Myeloid Leukemia

Acute myeloid leukemia (AML) encompasses a broad and diverse group of aggressive blood cell tumors arising from the uncontrolled spread of tumor hematopoietic precursor cells in the bone marrow.

AML is the most common acute leukemia in adult individuals (80% of acute leukemia) with a median age of 68 years at diagnosis [1]. Five-year relative survival after diagnosis of AML is estimated at 18% in Europe [1]. In Spain, the five-year relative survival has been estimated to be around 20% [2]. This survival significantly depends on the age of the patient (being approximately 6% in patients over 60 years of age, compared to 50% in younger patients), and there has been no notable improvement in recent years [2,3]. In addition, there is high toxicity associated with chemotherapy and high rates of relapse and resistance in elderly patients [4].

Leukemic cells often contain distinct sets of cytogenetic abnormalities and somatic mutations, resulting in considerable genetic complexity [5]. Clonal evolution within each AML patient appears to be a dynamic process with a continuous acquisition and loss of specific mutations. Exposure to chemotherapy regimens places enormous stress on AML cell populations and may be more toxic to certain cell clones than others. It has been observed that the clonal composition of AML change markedly after therapy in relapsed disease, with selection occurring at both the genetic and epigenetic levels [6].

Prognostic factors can be subdivided into those related to the patient and those related to the disease. Patient-related factors (e.g., advanced age, coexisting diseases or poor performance status) usually predict early death, whereas disease-related factors (e.g., prior myelodysplastic syndrome, prior exposure to cytotoxic or radiation therapy or cytogenetic and/or molecular findings in leukemic cells) predict resistance to current standard therapy or early relapse.

## 2. Role of Pharmacogenetics in the Treatment of Acute Myeloid Leukemia

First-choice treatment in healthy young adults consists of intensive chemotherapy induction followed by one or more consolidation cycles. The classic intensive chemotherapy regimen has been used for decades and consists of a combination of three days of anthracyclines (daunorubicin or idarubicin) and seven days of cytarabine, commonly referred to as 3 + 7. The complete remission rate in patients younger than 60–65 is around 60–80% [7]. After intensive therapy, one or more courses of high dose cytarabine is administered to eliminate residual disease and, if possible, proceed with hematopoietic stem cell transplantation (auotologous or allogeneic).

This classical treatment can be complemented by a more novel therapy consisting of the administration of drugs that target specific cancer cell receptors [8]: fms related receptor tyrosine kinase 3 (FLT3) inhibitors (midostaurin), isocitrate dehydrogenase (IDH) inhibitors (ivosidenib or enasidenib), or CD33 inhibitors (gemtuzumab ozogamicin).

Treatment of AML in elderly patients is based on the patients’ characteristics. The first stage is to determine medical fitness to make sound decisions about care and choice of treatment [9]. Although age is not the only determining factor, elderly patients are more likely to have comorbidities and to have a worse general condition, which makes them more prone to have complications in treatment with induction therapy [4]. Therefore, they are considered unfit for intensive chemotherapy.

It is important to highlight that there is no consensus on medical suitability criteria and there is no upper age limit. However, caution should be exercised when considering classical intensive therapy for patients older than 75 years, as most of the outcome data related to intensive antileukemic therapy come from studies in younger and fitter adults [10]. Treatment selection for these patients contemplates hypomethylating agents (decitabine or azacitidine), low-dose cytarabine, and, when possible, BCL2 apoptosis regulator (BCL2) inhibitors (venetoclax) or IDH inhibitors [11].

Advances in recent decades in the identification and analysis of certain cytogenetic and molecular alterations have made it possible to define different subgroups and entities with highly variable prognosis [12] leading to targeted therapy depending on the patient’s characteristics [13].

Treatment of AML should always be approached with the goal of curing the disease completely whenever possible. That said, classical induction therapy involves interindividual differences in treatment outcome and considerable toxicity, with a long-term survival rate of 35–45% in those under 60 years of age and 10–15% in those over 60 years of age [14]. It is possible that this interindividual variability in response and toxicity in AML patients treated with intensive chemotherapy may be due in part to genetic variability [7].

The implementation of precision medicine faces many challenges, one of them being the knowledge gap between disease genetics (somatic mutation) and pharmacogenetics (germline mutation) [15]. Disease genetics focuses on the knowledge of those genetic variants that are relevant for their pathogenicity or for their assistance in the management of some pathologies and treatment options. Pharmacogenetics is the branch of genetics that addresses the influence of genetic variations into drug response. Both disciplines are key pieces of precision medicine and should be valued together to improve clinical care, from disease diagnosis (related to disease genetics) to drug prescription (related to pharmacogenetics) [15].

Some frequently mutated genes in AML have been identified that are predictive of response to treatment, (e.g., *DNMT3A*, *ASXL1*, *TET2*, *IDH1*, *IDH2*, *NPM1*, *FLT3*, *RAS*, *RUNX1*, *ASXL1*, and *TP53*) [16] and, therefore, help clinicians to choose which therapeutic option might be the most appropriate. However, the implementation of pharmacogenetic analysis in routine clinical practice remains slow and ineffective. Therefore, a comprehensive review of the latest advances in the treatment of AML and the influence of patients’ genetic polymorphisms is needed to help shed light on the individualized management of these and other drugs used in AML [17].

The aim of this study is to carry out a review of the available publications and evidence related to the pharmacogenetics of AML, with the objective of compiling those studies that may be useful in optimizing drug administration in this type of patients.

**Figure 1:**
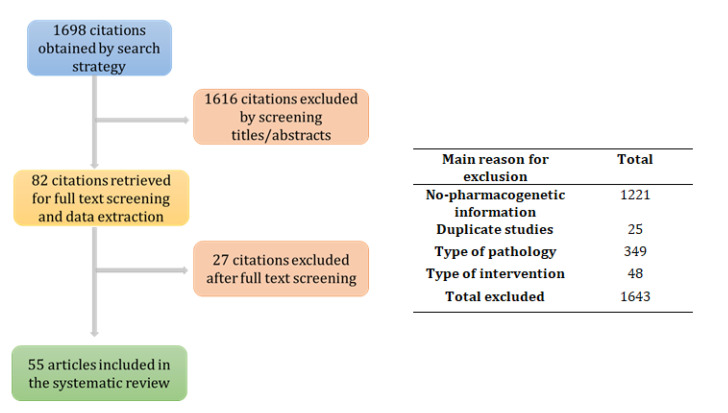
Flowchart of study selection procedure and main reasons for article exclusion.

This systematic review was conducted according to the Preferred Reporting Items for Systematic Reviews and Meta-analyses (PRISMA) statements [18]. The literature search included the following databases: MEDLINE, PharmGKB [19], EU Clinical Trials Register and ClinicalTrials.gov. The search strategy included similar keywords in all databases: acute myeloid leukemia, polymorphism (or SNP, or single nucleotide polymorphism or pharmacogenetics or pharmacogenomics), cytarabine, anthracyclines (idarubicin, daunorubicin), FLT3 inhibitors (midostaurin, gilteritinib), IDH inhibitors (ivosidenib, enasidenib) or CD33 inhibitors (gemtuzumab ozogamicin) or hypomethylating agents (azacitidine, decitabine) or BCL2 inhibitors (venetoclax). Two independent reviewers (APM and MSR) independently screened all of the titles and abstracts and evaluated each article. Disagreements were resolved by consensus and in concordance with a third reviewer (JL), when necessary. Studies that fulfilled the following criteria were included: (1) studies with pharmacogenetic biomarkers analysed in diagnosed AML patients’ cohorts in which any of the following were prescribed: cytarabine, idarubicin, daunorubicin, midostaurin, gilteritinib, ivosidenib, enasidenib, gemtuzumab ozogamicin, azacitidine, decitabine, or venetoclax; and (2) studies reporting efficacy and outcome variables. The systematic search retrieved 1698 citations, before removal of duplicates, these being the most relevant included in our review. The PRISMA flow chart of the selection procedure and main reasons for exclusion are detailed in Figure 1.

The following is a more detailed description of the drugs used in the treatment of AML, as well as the most relevant pharmacogenetic markers found for the different pharmacological agents.

## 3. Cytarabine

Cytarabine (ara-C) is an antineoplastic agent indicated for initial induction and maintenance therapy in AML. Ara-C is mainly metabolized in the liver, but also in the kidneys, in the gastrointestinal tract mucosa, in granulocytes, and to a lesser extent in other tissues. Deoxycytidine kinase (DCK) metabolizes ara-C to cytarabine monophosphate (ara-CMP), which is converted into cytarabine diphosphate (ara-CDP) by cytidine/uridine monophosphate kinase 1 (CMPK1) and into cytarabine triphosphate (ara-CTP) by nucleoside diphosphate kinase 1 (NME1) [20]. Although the mechanism of action is not fully known, the drug appears to inhibit DNA polymerase. Ara-CTP is a competitor of deoxycytidine 5′-triphosphate, which is a physiological substrate of DNA polymerase and therefore acts by inhibiting DNA synthesis. Moreover, cytidine deaminase (CDA) converts ara-C to the inactive metabolite uracil arabinoside (ara-U) [21], therefore, limiting the amount of ara-C to be converted to ara-CTP. The cytosolic nucleotidase enzymes NT5C2 and NT5C3A act oppositely, reactivating ara-CMP to ara-C [20].

Regarding DCK, several polymorphisms were associated with differences in treatment response in AML patients. DCK c.72C>T (rs377182313) and c.-201C>T (rs2306744) wild-type haplotype (C-C) was associated with poorer 2-year event free survival, whereas g.4949C>G (rs80143932) and c.-201C>T (rs2306744) variant alleles were associated with increased response [22]. DCK c.72C>T (rs377182313) and c.-201C>T (rs2306744) T alleles were also associated with higher survival rates in a Hispanic pediatric population [23]. DCK g.39600C>T (rs4694362) T allele was associated with higher overall survival [24], while c.*573T>C (rs72552079) and g.31006443A>C (rs11543896) C alleles were related to complete remission [25]. Moreover, c.*165C>T (rs4643786) heterozygotes showed higher complete remission rate and overall survival compared to wild-type individuals [26].

Since CDA is the key inactivating enzyme in cytarabine metabolic pathway, its overexpression is commonly associated with treatment resistance and relapse [27]. Three CDA polymorphisms (c.79A>C (rs2072671), c.208G>A (rs60369023) and c.435C>T (rs1048977)) were associated with reduced enzyme activity [28,29,30]. Moreover, the haplotype A-C-C defined by c.92A>G (rs602950), c.451C>T (rs532545) and c.897C>A (rs10916823) was related to higher enzymatic activity [31].

Several CDA polymorphisms were related to cytarabine toxicity and response, summarized in the study by Megías-Vericat et al. [20]. Patients with de novo AML and variant alleles of DCK c.-201C>T (rs2306744) and CDA c.-92A>G (rs602950) showed higher complete remission rates, along with lower survival rates for variant alleles of CDA c.79A>C (rs2072671), c.-31del (rs3215400) and wild-type genotype of c.-92A>G (rs602950) [32]. The study by Ciccolini et al. demonstrated that CDA deficiency lead to a higher risk of developing early severe toxicities with gemcitabine [33], another CDA substrate. A similar consequence might be expected in poor CDA metabolizer status patients, although this hypothesis has not yet been demonstrated.

5′-nucleotidase, cytosolic II (NT5C2) enzyme is expressed in all human tissues and acts opposite DCK by dephosphorylating ara-CMP, therefore limiting the production of ara-CTP [34,35]. Its overexpression is linked to cytarabine resistance and reduced survival rates in AML patients [36,37]. Variant alleles of NT5C2 c.7A>C (rs10883841), c.*6211C>T (rs11191547), c.*9860C>T (rs11191549), g.104851396G>T (rs11191553), c.*113+4808A>G (rs10883836) and g.3269C>T (rs7095304) were associated with lower disease-free survival in Caucasians [38]. For g.103261506G>A (rs1163075), the ancestral allele demonstrated higher NT5C2 expression levels and was associated with ara-C cytotoxicity [39]. Wild-type homozygous of NT5C2 promoter polymorphism g.60079A>C (rs11598702) showed significantly lower gemcitabine clearance compared to variant allele carriers [40]. Another member of this family is 5′-nucleotidase, cytosolic III (NT5C3), whose variant allele of g.33021334A>C (rs3750117) showed decreased expression and activity [41]. Only two studies evaluated the influence of NT5C3 polymorphisms in cytarabine response, finding no significant results for most of the alleles analyzed [24,42]. Only NT5C3 g.33021334A>C (rs3750117) variant allele resulted associated to higher expression and lower complete remission rates [42]. Further research is warranted to shed light on this controversy.

Cytarabine influx is mainly mediated by human equilibrative nucleoside transporter-1 (hENT1) [43], encoded by SCL29A1. When administered at the doses used in induction therapy (200 mg/m^2^), ara-C intracellular concentration depends mostly on hENT1 uptake [20]. The expression of hENT1 at diagnosis was proposed as a resistance mechanism to ara-C in AML patients, since hENT1 deficiency lead to shorter disease-free survival and overall survival [44].

SLC29A1 c.607G>C (rs61758845) C allele was associated with lower response to the gemcitabine-containing chemotherapy in lung cancer patients [45]. c.607G>C (rs61758845) showed a frequency of 21% in whites and 5% in African-Americans and might alter transcription factor binding sites [46]. Regarding AML, all of the available studies were performed in Asian population, given the higher frequencies of SLC29A1 polymorphisms minor alleles. SLC29A1 c.*469C>A (rs3734703) was significantly associated with higher complete remission rates [47]. Moreover, the haplotype A-G-G-T-C-A comprising g.4149T>G (rs507964), g.9679A>C (rs693955), g.12104G>C (rs747199), g.13751C>T (rs9394992), g.14337T>C (rs324148) and c.*469C>A (rs3734703) polymorphisms also revealed a significant association with complete remission [47]. Variant alleles of SLC29A1 g.13751C>T (rs9394992) and g.14337T>C (rs324148) were associated to lower overall survival, disease-free survival and higher relapse rate [48]. Likewise, the combination of SLC29A1 c.*469C>A (rs3734703) and thymidylate synthetase (TYMS) g.672363G>A (rs2612100) was significantly associated with shorter relapse free survival and decreased overall survival (although not statistically significant after multiple correction) [24]. Thymidine triphosphate generated by TYMS is known to enhance ara-C cytotoxicity by decreasing the amount of deoxycytidine triphosphate [49]. Therefore, although further research is needed, genetic variations of SLC29A1 seemed a potential marker of AML response when treated with ara-C.

Efflux mechanisms such as the activity of the P-glycoprotein (P-gp) transporter are importantly involved in mediating the multidrug resistance phenotype during cancer chemotherapy. P-gp is encoded by the *ABCB1* gene and has been commonly studied in AML. Several *ABCB1* polymorphisms have been associated with cytarabine response. AML patients with ABCB1 c.3435C>T (rs1045642) T/T genotype may have increased response to cytarabine regimens as compared to patients with the wild-type genotype [50], however the evidence is highly contradictory. Moreover, patients with ABCB1 c.1236C>T (rs1128503) T/T genotype may show a better response when treated with cytarabine, alone or in combination with daunorubicin, or dexrazoxane [51]. However, some evidence contradicts this. Besides, patients with homozygous mutated genotype for ABCB1 c.2677G>T/A (rs2032582) who are treated with cytarabine, idarubicin, or cytrarabine, daunorubicin and dexrazoxane may have an increased response [50]. Again, some contradictory evidence exists for these associations.

Finally, the RRM1 and RRM2 genes encode for ribonucleotide reductase subunits, which catalyzes the reduction of cytidine diphosphate into deoxycytidine triphosphate that enters the nucleus to block DNA synthesis [52]. Ribonucleotide reductase controls the amount of available deoxycytidine triphosphate, so its activity is directly related to sensitivity or resistance to cytarabine. A study in a AML pediatric cohort found the variant alleles of RRM1 c.*367G>A (rs1561876), c.*151A>T (rs1042919) and c.1138-52A>C (rs2898950), RRM2 c.-6T>C (rs1130609) and c.180C>G (rs5030743) and RRM2B g.102187808A>G (rs1265138) to be associated with a decreased response, poorer event-free survival and decreased overall survival [53]. Moreover, variant alleles of RRM1 g.4147113A>C (rs4593998), c.19+2487T>A (rs7130539), n.281A>C (rs11031136) and c.109-1267T>G (rs2268166) and RRM2B c.218-2602T>G (rs2853229) and c.1371+1077A>G (rs2607662) showed lower disease-free survival in Caucasians [38].

The metabolic pathway of cytarabine and the genes involved can be found in the study by Megías-Vericat et al. (2016) [20]. Regarding pharmacogenetic information, the clinical annotations found for cytarabine are categorized as level 3 of evidence, which include variant-drug combinations with a low level of evidence supporting the association [19]. All registered variants are summarized in Table 1.

## 4. Anthracyclines

Anthracyclines act trough the intrinsic mitochondrial pathways to exert their cytotoxic effects. They are metabolized in various tissues and cause DNA cleavage, interfering with replication, and generating reactive oxygen species (ROS). Genes of the ABC (ATP-binding cassette) and SLC (solute carrier) families are involved in the transport of the drug into the cell. Metabolism is carried out by oxidoreductases (nitric oxide synthase—NOS) and carbonyl reductases (CBR). The metabolic pathway of anthracyclines and the genes involved can be found in the study by Megías-Vericat et al. (2016) [58]. Regarding their safety profile, anthracycline-induced cardiotoxicity is a common, complex and devastating adverse reaction associated with significant morbidity and mortality [58].

Polymorphisms in membrane transporters (efflux pumps) of the ABC family have been studied in depth and have been associated with decreased transporter function and thus increased toxicity [59]. Several studies analyzed the influence of *ABCB1* polymorphisms in AML patients treated with anthracyclines, finding conflicting results, as some studies showed higher response or survival rates in patients carrying some variant alleles [51,60,61,62]; in contrast, other works did not confirm or even contradicted these associations [50,63,64,65,66]. To shed light on this controversy, a meta-analysis evaluated the influence of *ABCB1* most relevant polymorphisms on the response to standard chemotherapy (cytarabine plus anthracyclines) for AML [67]. This study reported higher overall survival among carriers of the variant alleles of *ABCB1* c.3435C>T (rs1045642), c.1236C>T (rs1128503) and c.2677G>TA (rs2032582). However, no influence on complete remission was demonstrated [67]. These results are suggestive of *ABCB1* polymorphisms in AML likely influencing the efficacy of standard chemotherapy, specifically in relation to OS, where the variants could have a positive effect due to lower P-gp expression. *ABCC1* polymorphisms seemed to be related to anthracycline-induced cardiotoxicity in childhood cancer [68] although these results were not confirmed in an AML cohort [65]. *ABCC1* g.16079375G>T (rs45511401) and *ABCC2* c.3563T>A (rs8187694) and c.4544G>A (rs8187710) influenced doxorubicin-induced cardiotoxicity in non-Hodgkin lymphoma [69]. Recently, these associations for *ABCC1* and *ABCC2* were not replicated in a 225 adult de novo AML patients cohort [70], but combinations of *SLCO1B1* and *ABCB1* polymorphisms were associated with higher toxicities [70]. Moreover, carriers of *ABCG2* c.34G>A (rs2231137) mutated allele showed higher survival but also increased odds for toxicity [65], which was confirmed in a subsequent study describing cardiac toxicity for the combination of *SLC22A16* c.146A>G (rs714368) and *ABCG2* c.421C>A (rs2231142) [70]. The presence of polymorphisms in ABC genes has been linked to treatment response and the progression of leukemia [71]. Consequently, they might be also potentially promising strategies to improve the treatment of AML patients with a multidrug resistance phenotype [71].

Regarding upstream transporters, OATP1B1, encoded by *SLCO1B1*, is predominantly expressed in the liver and is associated with plasma clearance of several drugs, including anthracyclines [72]. The mutated allele of the g.21331549T>C (rs4149056) polymorphism has been correlated with lower hepatic uptake of anthracyclines, increasing plasma levels and the risk of tissue toxicity and hepatotoxicity [73,74]. The wild-type genotype for g.64584249C>G (rs528211), g.124T>C (rs2360872), g.3791T>C (rs505802), c.-312C>G (rs524023), c.-287+21C>G (rs9734313), c.-83T>C (rs11231825) and c.1115-2898G>T (rs11606370) of the *SLC22A12* transporter was associated with poorer on-treatment survival [38]. 

Three pathways are involved in anthracycline metabolism: two-electron reduction (CBR and AKR enzymes), one-electron reduction (NQO1, xanthine oxidase or XDH and NOS1, NOS2 and NOS3) and deglycosylation (in addition to NQO1 and XDH the flavoprotein POR is involved). CBR1 and CBR3 are the proteins most related to anthracycline reduction reactions. Mutated alleles of *CBR1* c.-203+8928C>T (rs1143663) and c.391C>T (rs41557318) polymorphisms showed a decrease in treatment efficacy between 20 and 40% [38]. Moreover, *CBR3* c.730G>A (rs1056892) mutated allele were associated with higher activity than wild-type [74], although in AML patients the influence of *CBR3* is unclear [75]. *XDH* c.837C>T (rs4407290) and c.794-415A>G (rs2236168) mutated alleles were associated with lower cardiotoxicity, probably related to lower XDH expression or activity [38]. In a pediatric cohort treated with doxorubicin, the mutated allele of *NOS3* c.276T>A (rs1799983) was associated with increased neurotoxicity and a protective effect on cardiotoxicity [76]. In patients treated with daunorubicin, the mutated alleles of *POR* c.-39+562A>G (rs286868177), c.*235-2660G>A (rs13240755), c.*235-1161C>T (rs4732513) and c.106-562G>A (rs6953065) were associated with increased cardiotoxicity [77].

These pharmacogenetic studies show that anthracycline entry into leukemic cells depends on genetic variants of efflux pumps. Moreover, genetic variability of the genes encoding enzymes involved in anthracycline metabolism (CBR, AKR, XDH, NOS3) could lead to lower enzymatic activity and higher cardiotoxic potential.

The clinical annotations found for anthracyclines are categorized as level 3 of evidence, which include variant-drug combinations with a low level of evidence supporting the association [19]. All registered variants are summarized in Table 2.

## 5. FLT3 Inhibitors

Mutations in the FLT3 gene result in cell activation leading to uncontrolled cell multiplication. These mutations are associated with a high risk of relapse and reduced survival. FLT3 inhibitors target mutated cells that promote AML leukemogenesis and proliferation that occur in about 30% of AML cases [80]. There are two types of *FLT3* mutations, the most frequent is internal tandem duplication (ITD) but there are also kinase domain point mutations (TKD), although they are less frequent [81].

High relapse rates have been observed in patients treated with first generation FLT3 inhibitors such as midostaurin due to TKD mutations. Second-generation inhibitors such as gilteritinib target both *FLT3* ITD and TKD mutations and therefore have a therapeutic advantage [82].

Regarding pharmacogenetic biomarkers, information is scarce. One study shows that midostaurin blocks the expression of the P-gp transporter, encoded by *ABCB1*, which has the ability to cause drug resistance, so patients with multidrug-resistant tumors could benefit from combined midostaurin therapy with standard treatment [83]. This gives way to the hypothesis that, if polymorphisms in *ABCB1* alter its expression [84], in patients carrying these mutations, the use of midostaurin could provide a benefit.

Moreover, gilteritinib is mainly metabolized by cytochrome P450 (CYP) *CYP3A4* isoform, whose activity is extremely variable due to induction and inhibition, genetic polymorphism, drug interactions and diet [85]. Therefore, further research is warranted related to the pharmacogenetic information of FLT3 inhibitors.

## 6. IDH Inhibitors

These drugs block the action of mutated forms of isocitrate dehydrogenase (IDH) that occur in 15–20% of patients [86]. The mutated forms of IDH produce elevated levels of D-2-hydroxyglutarate (D2HG), which contributes to cancer cell growth. Ivosidenib blocks the action of mutated IDH1 and enasidenib blocks the mutated form IDH2 slowing disease progression [87,88].

Promising response rates have been observed in patients who received IDH inhibitors monotherapy for a prolonged time without incurring severe cytopenias, characteristic of intensively treated chemotherapy [89].

As for pharmacogenetic markers, the available information is scarce. A single clinical case links the mutated IDH genotype of c.394C>A (rs121913499) with resistance to ivosidenib treatment [90]. This association should be confirmed in studies with larger sample size.

## 7. CD33 Inhibitors

Gemtuzumab ozogamicin (GO) is a conjugate of anti-CD33 antibody and the cytotoxin calicheamicin that targets the surface antigen CD33. Increased cell surface expression of CD33 has been observed in patients with AML. In addition, CD33 expression levels have been correlated with disease characteristics such as the presence of FLT3-ITD [91].

Although GO is effective in some patients, the importance of evaluating the association between *CD33* genotypes of the most significant SNPs such as c.205A>G (rs2455069), c.529G>A (rs35112940), c.532T>C (rs61736475), c.*201G>C (rs1803254), c.466_469del (rs201074739) and CD33 cell surface expression levels in AML patients is noted to obtain a prediction of patient response on GO treatment [92].

Although the association between CD33 expression and clinical outcome is not fully defined, different genetic variants have been associated with different drug responses. In c.*201G>C (rs1803254), the CC+CG genotypes were associated with increased mortality and in c.529G>A (rs35112940) the CG genotype with increased survival compared to AA+AG genotypes [93]. *CD33* polymorphisms could serve to aid in the selection of suitable patients for GO therapies and possibly other CD33-targeted immunotoxins in future prospective trials.

In another study, alcohol dehydrogenase 1A (*ADH1A*) polymorphisms n.1039-3713C>T (rs1826909) and n.167-305G>A (rs6811453) were associated with reduced treatment efficacy. In addition, solute carrier organic anion transporter family member 1B1 (*SLCO1B1*) c.521T>C (rs4149056) and *CYP2E1* c.*549C>A (rs2515641) and c.-40+190A>T (rs2070673) were linked with abnormal renal function and increased renal toxicity of GO treatment [56].

Higher expression level of P-gp, encoded by *ABCB1*, constitutes a poor prognostic factor related to overall survival and event-free survival [94,95]. In addition, CD33 expression was inversely correlated with P-gp drug efflux activity [95]. *ABCB1* c.3435C>T (rs1045642) might alter P-gp activity [96], although there is still controversy whether the mutated T allele is associated with a reduced functionality of P-gp [97]. A recent study revealed that patients under GO treatment carriers of the T allele showed improved outcomes compared to wild-type individuals [98]. The fact that *ABCB1* polymorphisms affect drug disposition leads to consider the analysis of *ABCB1* haplotypes instead of individual variants [97]. Therefore, once validated, this *ABCB1* polymorphisms association together with *CD33* polymorphisms may open up opportunities to personalize GO-therapy [98].

## 8. Hypomethylating Agents

Hypomethylating agents act on aberrant hypermethylation of tumor suppressor genes. These genes are involved in normal cell cycle regulation, differentiation and apoptosis pathways. Hypomethylation can restore the function of tumor suppressor genes in cancer cells. Azacitidine and decitabine are used indistinctly, depending on the preference of the practitioner, as the superiority of either has not yet been demonstrated [99].

Azacitidine is metabolized by cytidine deaminase, encoded by the highly polymorphic CDA gene. A deficiency in the CDA gene has been associated with resistance and increased toxicity to treatment with azacitidine [100,101]. A single-case report of an AML patient with CDA c.79A>C (rs2072671) mutant homozygous genotype with a marked deficient phenotype showed life-threatening toxicities after a single cycle of azacitidine [100]. Moreover, CDA poor-metabolizer phenotype suggested that an impaired detoxification step could have given rise to the lethal toxicities observed in a azacitidine-treated patient [101]. Further prospective studies exploring the exact role of CDA metabolizer status on the clinical outcome of patients treated with azacitidine are warranted.

Thymidylate synthase (TS) catalyzes the conversion of deoxyuridine monophosphate to deoxyxymidine monophosphate using a substrate of the enzyme methylenetetrahydrofolate reductase (MTHFR) during DNA synthesis and repair. It has been shown that TS may be involved in the etiopathogenesis of AML. MTHFR c.*177C>G (rs1801133), TS c.*450_*455del (rs11280056) and X-ray repair cross complementing 1 (XRCC1) (DNA repair protein) c.*310A>C (rs25487) mutated alleles have been associated with shorter survival in patients with myelodysplastic syndromes treated with azacitidine versus patients who only received supportive care [102]. This associated should also be evaluated in AML patients.

Increased expression of TBC1 domain family member 16 (TBC1D16), a gene involved in the regulation of cell growth and differentiation, is associated with an increased risk of non-remission and worse overall survival in patients with AML. In contrast, blockade of the TBC1D16 gene results in decreased cell proliferation and phosphorylation levels, as well as increased sensitivity to decitabine. When treated with decitabine, patients who achieve complete remission show significantly lower TBC1D16 expression. Conversely, blockade of TBC1D16 results in decreased sensitivity to cytarabine. These findings imply that TBC1D16 is a potential predictor of chemosensitivity and prognosis in adult AML patients [103] and should be considered as a potential pharmacogenetic biomarker.

## 9. BCL2 Inhibitors

Venetoclax is a selective inhibitor of the broad-spectrum anti-apoptotic protein BCL2 and has shown cytotoxic activity in BCL2-overexpressing tumor cells. Although not considered a targeted therapy, the inhibitory effect of venetoclax preferentially targets IDH-mutated AML cells [104]. In IDH-expressing patients, the therapy response of enasidenib (IDH inhibitor) is enhanced in combination with venetoclax due to enasidenib-induced differentiation and reduced anti-apoptotic protein expression due to venetoclax [105].

In vitro characterization showed that venetoclax is mainly metabolized by CYP3A4, consistent with drug-drug interaction studies performed with strong CYP3A4 inhibitors [106,107]. Moreover, venetoclax is also a substrate of P-gp [108]. Therefore, further pharmacogenetics approaches should include *CYP3A* and *ABCB1* as candidate genes to assess.

### Other Considerations

Antifungal prophylaxis, such as posaconazole, which is metabolized by the CYP3A4 isoform of cytochrome P450, is very common in the treatment of leukemia. This enzyme in turn metabolizes multiple drugs, including midostaurin. Thus, concomitant administration of both drugs leads to the accumulation of midostaurin that is not being metabolized, requiring dose adjustment based on concomitant treatments [109].

In all cases, the aim of both the use of pharmacogenetics and the selection of concomitant drugs is to individualize treatment. In this way, the drug that a priori will be most effective for the patient is selected, avoiding possible adverse reactions that may occur due to a decrease in drug metabolism (caused by genetic mutations) or by blockage of the metabolizing enzyme (caused by concomitant medication).

## 10. Future Perspectives

AML remains a difficult-to-treat disease in need of novel and effective therapies. The long-term goal of pharmacogenetic studies is to use genotype data to predict the effective treatment response to a specific drug and in turn prevent potential undesirable effects of its administration. Pharmacogenetic information is useful to optimize AML therapy by selecting in each case the most appropriate treatment based on the genetic characteristics of both disease and patient.

Most of the results described in this review were obtained from retrospective observational cohort studies, without validation of these particular findings. Further approaches undoubtedly should test prespecified hypotheses in prospective randomized clinical trials in larger populations. Another issue to bear in mind is the limitations of the candidate-gene approach, which preclude the identification of causal variants that are not included in the analysis. It is expected that the evolution of genome sequencing techniques will improve access to pharmacogenetic information on patients and diseases in the coming years, in order to guide the development and use of new therapies that improve response to treatment, increase its effectiveness and reduce toxicity.

Although studies linking treatment outcome to the expression of specific genes in AML are presented, further understanding of the disease and the pharmacogenetics of available therapies is still needed. In the studies reviewed, cohorts are usually small and protocols are not standardized. In addition, the tests are not selective and the results obtained are difficult to interpret. Most of the studies found refer to the classic and most widely used treatments, but there is not yet enough relevant data available for the newer treatments. Given the limited level of evidence, recommendations on the performance of a pharmacogenetic test prior to treatment is a premature approach that we cannot embrace at this moment. These limitations should be addressed in future studies so that clinical application is properly supported, feasible and applicable.

The relevance of polymorphisms in treatment response needs to be strongly confirmed so that the results can be applied to routine clinical practice, with the aim of optimizing drug delivery in AML patients.

It is also necessary to establish early genotyping strategies and demonstrate their utility in randomized clinical trials, in order to assess whether genotyping optimizes responses and reduces the rate of adverse reactions.

According to our knowledge, no specific pharmacogenetic biomarkers gene panel has been developed so far. However, several next-generation sequencing panels specialized in somatic mutations have been settled, including a large amount of genes (e.g., *CREBBP*, *DNAH9*, *POU2F2*, *GNB1*, *BCORL1*, *GNAS*, *CNOT3*, *JAK2*, *STAG2*, *ELF4*, *MAZ*, *EZH2*, *CA9*, *SMC3*, *SHOC2*, *CBL*, *LTA4H*, *SF3B1*, *SOS1*, *MPL*, *STAG1*, *KMT2A*, *CSF3R*, *DNMT3A*, *FLT3*, *MAP1B*, *RAF1*, *KRAS*, *IDH1*, *TP53*, *SETDB1*, *RIT1*, *NOTCH1*, *POU4F1*, *SETBP1*, *FMN2*, *PHF6*, *KIT*, *BRAF*, *RUNX1*, *U2AF1*, *SRSF2*, *TET2*, *MAP2K1*, *ZRSR2*, *AKAP13*, *PPM1D*, *ASXL1*, *MYD88*, *CALR*, *PTPN11*, *NPM1*, *SETD2*, *IDH2*, *BCOR*, *WT1*, *NF1*, *HTT*, *NRAS*, *TLR9*, *CEBPA*, and *CUX1* (Ref: LV3882, © Sistemas Genómicos, Valencia, Spain). Once the pharmacogenetic associations depicted in this review are consolidated as reliable biomarkers, similar approaches might be implemented. A broad pharmacogenetic knowledge of the entire therapeutic arsenal can be of great use in designing dosing strategies adapted to the genotype or phenotype of the disease and the patient and, consequently, can be used as variables in AML treatment guidelines.

It would be worthwhile to perform a broad pharmacogenetic study that evaluates the response to the drugs mainly used for the treatment of AML described in this review. The most appropriate approach would be a genome-wide association study (GWAS) in a large number of patients, as it may allow the discovery of genes and variants related to drug effect that had not been previously described, without the need for exhaustive knowledge of the physiology of the disease or the pharmacokinetics or pharmacodynamics of the drug. However, this strategy is not accessible to all research groups, since the costs are higher than the analysis using candidate genes. In the event that the latter is the methodology of choice, to help decide which genetic variants would be interesting to analyze we have reflected in Table 3 the most relevant ones.

## Figures and Tables

**Table 1:** Summary of the pharmacogenetic associations related to cytarabine response in AML patients.

Gene	Variant	Genotype	Number of Patients	Ethnicity	MAF in European Population	Association	Reference (Type of Study)
*ABCB1*	rs2032582 (G>A) c.2677G>T/A	A/A	101	Asian population (Korea)	0.41 (A) 0.01 (T)	Increased response	Kim et al., 2006 [50] Prospective
	rs1045642 (C>T) c.3435T>A	T/T	101	Asian population (Korea)	0.48 (C)	Increased response	Kim et al., 2006 [50] Prospective
	rs1128503 (C>T) c.1236T>C	T/T	100	European population (Sweden)	0.42 (T)	Increased response	Gréen et al., 2012 [51] Prospective
*CDA*	rs2072671 (A>C) c.79A>C	C/C	100	South Asian population (India)	0.31 (C)	Increased toxicity	Abraham et al., 2012 [30] Prospective
	rs602950 (A>G) c.-92A>G	G/G	100	South Asian population (India)	0.30 (G)	Increased toxicity	Abraham et al., 2012 [30] Prospective
	rs532545 (C>T) g.20588679C>T	T/T	360	European population (Germany)	0.30 (T)	Decreased survival time and increased risk of death	Mahlknecht et al., 2009 [54] Prospective
	rs60369023 (G>A) c.208G>A	A/A	NA	NA	0.00 (A)	Decreased catalytic activity (in vitro)	Baker et al., 2013 [55]
*DCK*	rs80143932 (C>G) g.4929C>G	G/G	122	Asian population (China)	0.01 (G)	Increased response	Shi et al., 2004 [22] Prospective
	rs2306744 (C>T) c.-201C>T	T/T	122	Asian population (China)	0.01 (T)	Increased response Poorer 2-year event free survival	Shi et al., 2004 [22] Prospective
	rs377182313 (C>T) c.72C>T	C/C	122	Asian population (China)	<0.01 (T)	Poorer 2-year event free survival	Shi et al., 2004 [22] Prospective
	rs4643786 (C>T) c.*165C>T	C/T	282	Asian population (China)	0.04 (C)	Higher complete remission rate and overall survival	Zhang et al., 2016 [26] Prospective
	rs377182313 (C>T) c.72C>T	T/T	27 pediatric patients	American population (Mexico)	0.00 (T)	Higher survival rates	Medina-Sanson et al., 2015 [23] Prospective
	rs2306744 (C>T) c.-201C>T	T/T	27 pediatric patients	American population (Mexico)	0.01 (T)	Higher survival rates	Medina-Sanson et al., 2015 [23] Prospective
	rs4694362 (C>T) g.39600C>T	T/T	97	Asian population (Korea)	0.51 (T)	Higher overall survival	Kim et al., 2013 [24] Prospective
	rs72552079 (T>C) c.*573T>C	C/C	151	Asian population (China)	0.00 (C)	Increased response	Xu et al., 2012 [25] Retrospective
	rs11543896 (C>A) g.31006443A>C	A/A	151	Asian population (China)	0.35 (A)	Decreased response	Xu et al., 2012 [25] Retrospective
*NT5C3*	rs3750117 (A>G) g.33021334A>G	G/G	103	Asian population (Korea)	0.29 (A)	Decreased response	Cheong et al., 2014 [42] Retrospective
*RRM1*	rs1561876 (A>G) c.*367G>A	G/G	90 + 90 pediatric patients	European + African populations	0.12 (G)	Decreased response	Cao et al., 2013 [53] Prospective
	rs2898950 (C>A) g.4104060A>C	A/A	90 + 90 pediatric patients	European + African populations	0.08 (C)	Decreased response	Cao et al., 2013 [53] Prospective
	rs1042919 (A>T) c.*151A>T	A/T	90 + 90 pediatric patients	European + African populations	0.07 (A)	Poorer event-free survival	Cao et al., 2013 [53] Prospective
	rs4593998 (G>A) g.4168343A>G	A/A	154 + 125	European + non-european (African, Asian and Mexican) populations	0.12 (A)	Lower disease-free survival	Yee et al., 2013 [38] Retrospective
	rs7130539 (T>C) g.4118748T>C	C/C	154 + 125	European + non-european (African, Asian and Mexican) populations	0.05 (C)	Lower disease-free survival	Yee et al., 2013 [38] Retrospective
	rs11031136 (T>G) g.4169663T>G	G/G	154 + 125	European + non-european (African, Asian and Mexican) populations	0.05 (G)	Lower disease-free survival	Yee et al., 2013 [38] Retrospective
	rs2268166 (T>G) g.4126009T>G	G/G	154 + 125	European + non-european (African, Asian and Mexican) populations	0.05 (G)	Lower disease-free survival	Yee et al., 2013 [38] Retrospective
*RRM2*	rs5030743 (C>G) c.330C>G	G/G	90 + 90 pediatric patients	European + African populations	0.00 (G)	Poorer overall survival	Cao et al., 2013 [53] Prospective
	rs1130609 (G>T) c.-6T>G	T/T	90 + 90 pediatric patients	European + African populations	0.27 (T)	Poorer overall survival	Cao et al., 2013 [53] Prospective
*RRM2B*	rs1265138 (A>G) g.103200036A>G	G/G	90 + 90 pediatric patients	European + African populations	0.05 (G)	Decreased response	Cao et al., 2013 [53] Prospective
*SLC22A12*	rs11231825 (T>C) c.-83T>C	C/C	94	European population (Italy)	0.29 (T)	Decreased likelihood of fever	Iacobucci I et al., 2013 [56] Prospective
*SLC29A1*	rs3734703 (C>A) c.*469C>A,	A/A	103	Asian population (Korea)	0.02 (A)	Higher complete remission rates	Kim et al., 2016 [47] Retrospective
	rs9394992 (C>T) g.44195992C>T	T/T	100	Asian population (China)	0.28 (T)	Lower overall survival and disease-free survival and higher relapse rate	Wan et al., 2014 [48] Retrospective
	rs324148 (C>T) g.44196578T>C	T/T	100	Asian population (China)	0.25 (T)	Lower overall survival and disease-free survival and higher relapse rate	Wan et al., 2014 [48] Retrospective
*SLC29A1* (haplotype)	rs507964 (G>T) g.44218653T>G	Haplotype T-C-G-T-C-A	103	Asian population (Korea)	0.37 (T)	Higher complete remission rates	Kim et al., 2016 [47] Retrospective
	rs693955 (C>A) c.-159+228A>C				0.18 (A)		
	rs747199 (G>C) g.44194345G>C				0.22 (C)		
	rs9394992 (C>T) g.44195992C>T				0.28 (T)		
	rs324148 (C>T) g.44196578T>C				0.25 (T)		
	rs3734703 (C>A) c.*469C>A				0.02 (A)		
*SLCO1B1*	rs4149056 (T>C) c.521T>C	C/C	94	European population (Italy)	0.16 (C)	Increased likelihood of toxic liver disease	Iacobucci I et al., 2013 [56] Prospective
	rs2291075 (T>C) c.597C>T	C/C	164 pediatric patients	Utah residents with ancestry from northern and western Europe)	0.40 (T)	Less favorable event-free and overall survival	Drenberg et al., 2016 [57] Prospective
*SULT2B1*	rs2302948 (C>T) c.592C>T	T/T	94	European population (Italy)	0.24 (T)	Decreased likelihood of fever	Iacobucci I et al., 2013 [56] Prospective
*TYMS*	rs2612100(G>A) g.672363G>A	A/A	97	Asian population (Korea)	0.33 (A)	In combination with SLC29A1 rs3734703 (C/A or A/A), shorter relapsed-free survival	Kim et al., 2013 [24] Prospective

Abbreviation: *ABCB1*, ATP binding cassette subfamily B member 1; *CDA*, cytidine deaminase; *CYP2E1*, cytochrome P450 family 2 subfamily E member 1; *DCK*, deoxycytidine kinase; MAF, minor allele frequency; *NTC5C3*, cytosolic 5′-nucleotidase-III; *RRM1*, ribonucleotide reductase catalytic subunit M1; *RRM2*, ribonucleotide reductase regulatory subunit M2; *RRM2B*, ribonucleotide reductase regulatory TP53 inducible subunit M2B; *SLC22A12*, solute carrier family 22 member 12; *SLC29A1*, solute carrier family 29 member 1, *SLCO1B1*, solute carrier organic anion transporter family member 1B1; *SULT2B1*, sulfotransferase family 2B member 1; *TYMS*, thymidylate synthetase.

**Table 2:** Summary of the pharmacogenetic associations related to anthracyclines (idarubicin and daunorubicin) response in AML patients.

Gen	Variant	Genotype	Number of Patients	Ethnicity	MAF in European Population	Association	Reference (Type of Study)
**Idarubicin**							
*ABCB1*	rs2032582 (G>A) c.2677G>T/A	A/A	101	Asian population (Korea)	0.41 (A) 0.01 (T)	Increased response	Kim et al., 2006 [50] Prospective
*CYBA*	rs4673 (C>T) c.214T>C	T/T	225	European population (Spain)	0.34 (T)	Lower overall survival	Megías-Vericat et al., 2017 [78] Retrospective
*NCF4*	rs1883112 (G>A) g.37256846G>A	A/A	225	European population (Spain)	0.42 (A)	Higher complete remission	Megías-Vericat et al., 2017 [78] Retrospective
*RAC2*	rs13058338 (T>A) g.37236730T>A	A/A	225	European population (Spain)	0.23 (A)	Higher complete remission	Megías-Vericat et al., 2017 [78] Retrospective
*SLC22A12*	rs11231825 (T>C) c.426T>C	C/C	94	European population (Italy)	0.29 (T)	Decreased likelihood of fever	Iacobucci I et al., 2013 [56] Prospective
*SLCO1B1*	rs4149056 (T>C) c.521T>C	C/C	94	European population (Italy)	0.16 (C)	Increased likelihood of toxic liver disease	Iacobucci I et al., 2013 [56] Prospective
*SULT2B1*	rs2302948 (C>T) c.592C>T	T/T	94	European population (Italy)	0.24 (T)	Decreased likelihood of fever	Iacobucci I et al., 2013 [56] Prospective
**Daunorubicin**							
*ABCB1*	rs2032582 (G>A) c.2677G>T/A	A/A	101	Asian population (Korea)	0.41 (A) 0.01 (T)	Increased response	Kim et al., 2006 [50] Prospective
*NOS3*	rs1799983 (G>T) c.894T>G	T/T	225	Asian population (China)	0.34 (T)	Decreased survival	He et al., 2014 [79] Retrospective
*SLCO1B1*	rs2291075 (T>C) c.597C>T	C/C	164 pediatric patients	Utah residents with ancestry from northern and western Europe)	0.40 (T)	Less favorable event-free and overall survival in children	Drenberg et al., 2016 [57] Prospective

Abbreviation: ABCB1, ATP binding cassette subfamily B member 1; *CYBA*, cytochrome b-245 alpha chain; *NCF4*, neutrophil cytosolic factor 4; *NOS3*, nitric oxide synthase 3; *RAC2*, Rac family small GTPase 2; *SLC22A12*, solute carrier family 22 member 12; *SLCO1B1*, solute carrier organic anion transporter family member 1B1; *SULT2B1*, sulfotransferase family 2B member 1.

**Table 3:** Summary of the variants of interest for the drugs analyzed in this review.

Gene	Variant	Drugs Related *
*ABCB1*	c.2677G>A rs2032582	Cytarabine, idarubicin, daunorubicin, gemtuzumab ozogamicin, venetoclax
	c.3435C>T rs1045642	Cytarabine, idarubicin, daunorubicin, gemtuzumab ozogamicin, venetoclax
	c.1236C>T rs1128503	Cytarabine, idarubicin, daunorubicin, gemtuzumab ozogamicin, venetoclax
*ADH1A*	n.1039-3713C>T rs1826909	Gemtuzumab ozogamicin
	n.167-305G>A rs6811453	Gemtuzumab ozogamicin
*CDA*	c.79A>C rs2072671	Cytarabine, azacitidine
	c.-92A>G rs602950	Cytarabine, azacitidine
	g.20588679C>T rs532545	Cytarabine, azacitidine
	c.208G>A rs60369023	Cytarabine, azacitidine
*CD33*	c.205A>G rs2455069	Gemtuzumab ozogamicin
	c.529G>A rs35112940	Gemtuzumab ozogamicin
	c.532T>C rs61736475	Gemtuzumab ozogamicin
	c.*201G>C rs1803254	Gemtuzumab ozogamicin
	c.466_469del rs201074739	Gemtuzumab ozogamicin
*CYBA*	c.214T>C rs4673	Idarubicin
*CYP2E1*	c.*549C>A rs2515641	Gemtuzumab ozogamicin
	c.-40+190A>T rs2070673	Gemtuzumab ozogamicin
*CYP3A4*	c.1011del rs67666821	Gilteritinib, midostaurin, venetoclax
	c.375-191C>T rs35599367	Gilteritinib, midostaurin, venetoclax
*DCK*	g.4929C>G rs80143932	Cytarabine
	c.-201C>T rs2306744	Cytarabine
	c.72C>T rs377182313	Cytarabine
	c.*165C>T rs4643786	Cytarabine
	c.72C>T rs377182313	Cytarabine
	c.-201C>T rs2306744	Cytarabine
	g.39600C>T rs4694362	Cytarabine
	c.*573T>C rs72552079	Cytarabine
	g.31006443A>C rs11543896	Cytarabine
*IDH*	*c.394C>A* rs121913499	Ivosidenib
*MTHFR*	c.*177C>G rs1801133	Azacitidine
*NCF4*	g.37256846G>A rs1883112	Idarubicin
*NOS3*	c.894T>G rs1799983	Daunorubicin
*NT5C3*	g.33021334A>G rs3750117	Cytarabine
*RAC2*	g.37236730T>A rs13058338	Idarubicin
*RRM1*	c.*367G>A rs1561876	Cytarabine
	g.4104060A>C rs2898950	Cytarabine
	c.*151A>T rs1042919	Cytarabine
	g.4168343A>G rs4593998	Cytarabine
	g.4118748T>C rs7130539	Cytarabine
	g.4169663T>G rs11031136	Cytarabine
	g.4126009T>G rs2268166	Cytarabine
*RRM2*	c.330C>G rs5030743	Cytarabine
	c.-6T>G rs1130609	Cytarabine
*RRM2B*	g.103200036A>G rs1265138	Cytarabine
*SLC22A12*	c.-83T>C rs11231825	Cytarabine, Idarubicin
*SLC29A1*	c.*469C>A rs3734703	Cytarabine
	g.44195992C>T rs9394992	Cytarabine
	g.44196578T>C rs324148	Cytarabine
*SLCO1B1*	c.521T>C rs4149056	Cytarabine, idarubicin, gemtuzumab ozogamicin
	c.597C>T rs2291075	Cytarabine, daunorubicin
*SULT2B1*	c.592C>T rs2302948	Cytarabine, idarubicin
*TS*	c.*450_*455del rs11280056	Azacitidine
*TYMS*	g.672363G>A rs2612100	Cytarabine
*XRCC1*	c.*310A>C rs25487	Azacitidine

* Note that the clinical annotations described in this review are mostly categorized as level 3 of evidence, which include variant-drug combinations with a low level of evidence supporting the association. Further research is warranted.

## Data Availability

Not applicable.
